# How do population, general practice and hospital factors influence ambulatory care sensitive admissions: a cross sectional study

**DOI:** 10.1186/s12875-017-0638-9

**Published:** 2017-05-25

**Authors:** John Busby, Sarah Purdy, William Hollingworth

**Affiliations:** 1 Centre for Public Health, Queen’s University Belfast, BT12 6BA Belfast, UK; 2 School of Social and Community Medicine, University of Bristol, BS8 2PS Bristol, UK

**Keywords:** Primary health care, General practice, Ambulatory care, Patient admission

## Abstract

**Background:**

Reducing unplanned hospital admissions is a key priority within the UK and other healthcare systems, however it remains uncertain how this can be achieved. This paper explores the relationship between unplanned ambulatory care sensitive condition (ACSC) admission rates and population, general practice and hospital characteristics. Additionally, we investigated if these factors had a differential impact across 28 conditions.

**Methods:**

We used the English Hospital Episode Statistics to calculate the number of unplanned ACSC hospital admissions for 28 conditions at 8,029 general practices during 2011/12. We used multilevel negative binomial regression to estimate the influence of population (deprivation), general practice (size, access, continuity, quality, A&E proximity) and hospital (bed availability, % day cases) characteristics on unplanned admission rates after adjusting for age, sex and chronic disease prevalence.

**Results:**

Practices in deprived areas (at the 90th centile) had 16% (95% confidence interval: 14 to 18) higher admission rates than those in affluent areas (10th centile). Practices with poorer care continuity (9%; 8 to 11), located closest to A&E (8%; 6 to 9), situated in areas with high inpatient bed availability (14%; 10 to 18) or in areas with a larger proportion of day case admissions (17%; 12 to 21) had more admissions. There were smaller associations for primary care access, clinical quality, and practice size. The strength of associations varied by ACSC. For example, deprivation was most strongly associated with alcohol related diseases and COPD admission rates, while continuity of primary care was most strongly associated with admission rates for chronic diseases such as hypertension and iron-deficiency anaemia.

**Conclusions:**

The drivers of unplanned ACSC admission rates are complex and include population, practice and hospital factors. The importance of these varies markedly across conditions suggesting that multifaceted interventions are required to avoid hospital admissions and reduce costs. Several of the most important drivers of admissions are largely beyond the control of GPs. However, strategies to improve primary care continuity and avoid unnecessary short-stay admissions could lead to improved efficiency.

**Electronic supplementary material:**

The online version of this article (doi:10.1186/s12875-017-0638-9) contains supplementary material, which is available to authorized users.

## Background

Unplanned admissions place a tremendous strain on UK healthcare resources, accounting for 67% of hospital bed days, costing £12.5bn annually [1] and disrupting elective care [2]. In England, they have increased by 47% over the last 15 years [1] with some arguing that their continued rise threatens to bankrupt the National Health Service (NHS) [3]. Reducing the number of unplanned admissions is a key priority [4], but is challenging due to complex hospital admission pathways.

Ambulatory care sensitive conditions (ACSCs) account for one in five unplanned admissions [5]. ACSCs are conditions where improved primary and community care could potentially prevent the need for hospital admission [6]. Substantial unexplained geographic variation in ACSC admission rates [7, 8] suggests reductions might be possible, however it remains uncertain how these can be achieved. Knowledge of how population (e.g. deprivation), general practice (e.g. quality) and hospital (e.g. bed availability) characteristics influence admission rates could aid the identification of poorer quality care, help redesign services, develop admission avoidance interventions, and yield financial savings for the NHS. However, currently this evidence is only available for a minority of ACSCs and is often impaired by poor generalisability, a lack of case-mix adjustment and other methodological weaknesses [9, 10].

In this paper we use routine data from English hospitals to examine the relationship between unplanned ACSC admission rates and several population, general practice and hospital characteristics. We determine if these factors have a differential impact across conditions.

## Methods

### Data source and preparation

Our study was set in the English NHS. The NHS is comprised of three broad sectors; primary care (e.g. general practitioners, pharmacists, community care), secondary care provided in hospitals, and specialist tertiary care. Primary care doctors are paid by the NHS though a weighted capitation method (based on the number and complexity of the patients under their care) with additional payments dependant on achieving quality targets through the Quality and Outcomes Framework (QOF). Hospital treatment is generally reimbursed by the NHS on a fixed payment system. Almost all services are free at the point of access.

We used the Hospital Episode Statistics (HES) admitted patient care dataset to identify admissions for 28 common (i.e. >3,000 admissions annually) ACSCs between 1st April 2011 and 31st March 2012 using ICD diagnosis codes from previous work (Additional file 1) [6]. We aggregated admissions to the level of the general practice and excluded practices with a registered population smaller than 1,000 (*n* = 68; 0.8%), as these are likely to represent atypical practices, or with missing covariate data (*n* = 26; 0.3%) leaving 8,029 practices, with over 55 million registered patients, in the final analysis. Practices were located within all 151 Primary Care Trusts (PCTs) that, at the time of the study, commissioned care for their local populations. We investigated the effect of population (deprivation), general practice (size, access, continuity, quality, A&E proximity) and hospital (bed availability, percentage day cases) characteristics on unplanned admission rates. Further details on the variables used within the analysis, including their potential weaknesses, are provided in Additional file 2.

### Association with practice and hospital characteristics

We described the demographics of patients admitted for each condition. We estimated the relationship between unplanned ACSC admission rates and population, practice and hospital characteristics using two-level multivariate negative binomial regression models. These models include random PCT-effects which appropriately account for the clustering of practices within PCTs, adjust for latent PCT-level characteristics which could affect the demand for hospital admission, and allow the association of practice-level (e.g. access) and PCT-level (e.g. inpatient bed availability) characteristics to be estimated simultaneously. Each independent variable was scaled by the difference between a high (90th centile) and low (10th centile) practice or PCT to improve comparability across covariates (see Additional file 2 for further details).

We adjusted for differences in practice populations using a two-step process. First, we calculated the expected number of admissions using indirect standardisation (using quinary age groups and gender) and national data [11] to account for differences in the size and age-sex composition of practice populations (Additional file 3). We used negative binomial regression, and data from the QOF [12], to adjust for disparities in chronic disease prevalence among practices (atrial fibrillation, asthma, cancer, CKD, COPD, dementia, epilepsy, heart failure, hypertension, learning disability, mental health problems, obesity and stroke). Exploratory analysis revealed a non-linear relationship with A&E proximity for several conditions, so this were modelled using quartiles.

## Results

### Descriptive statistics

There were 1.77 million admissions for ACSCs accounting for 10.9 million bed days during 2011/2 (Table 1). Many admitted patients were older (mean age 56 years old), lived in more deprived communities (27% lowest quintile), had at least one comorbidity recorded (58%) and were admitted through A&E (75%). These overall results concealed substantial variation between conditions (Table 2), for example, over 30% of iron-deficiency anaemia and ENT infection admissions originated from a GP referral, while this was the case for less than 5% of schizophrenia and alcohol-related disease admissions.

**Table 1:** Admission details for all ACSCs admissions

Characteristics	Count (%)
Number of Admissions	1,767,550
Bed Days	10,903,662
Day Cases	443,760 (25.1)
Mean Age	55.6
0–19	264,541 (15.0)
20–39	207,032 (11.7)
40–59	338,316 (19.1)
60–79	512,017 (29.0)
80+	445,644 (25.2)
Male	844,537 (47.8)
Ethnicity	
White	1,495,974 (84.6)
Asian	100,486 (5.7)
Black	41,879 (2.4)
Mixed	14,623 (0.8)
Missing	114,588 (6.5)
Deprivation	
0 (Most Deprived)	477,437 (27.0)
1	387,099 (21.9)
2	339,554 (19.2)
3	302,310 (17.1)
4 (Least Deprived)	261,150 (14.8)
Comorbidities	
Any	1,032,628 (58.4)
Chronic obstructive pulmonary disease	465,731 (26.4)
Diabetes	288,168 (16.3)
Congestive heart failure	194,692 (11.0)
Cerebrovascular disease	185,824 (10.5)
Renal disease	133,975 (7.6)
Admission Source	
The usual place of residence	1,671,614 (94.6)
Other	94,806 (5.4)
Admission Method	
Emergency: via accident and emergency	1,326,882 (75.1)
Emergency: via general practitioner	290,218 (16.4)
Other	150,450 (8.5)
Discharge Destination	
The usual place of residence	1,597,060 (90.4)
Patient died	80,371 (4.6)
Nursing home	35,425 (2.0)
Other	54,694 (3.1)

**Table 2:** Characteristics of admitted patients by condition

Condition	Mean Age	% Male	% Most Deprived Quintile	% Admitted From GP	% Day Case
Alcohol-related diseases	43.8	68.0	40.1	4.3	35.4
Angina	60.5	54.6	27.0	9.1	37.8
Asthma	31.2	42.9	32.9	16.1	27.1
Atrial fibrillation / flutter	56.3	40.7	21.4	12.6	41.0
Cellulitis	51.6	52.1	27.4	26.6	22.5
Congestive heart failure	78.5	51.6	22.9	19.8	6.9
Constipation	51.4	42.6	27.6	27.0	32.4
Convulsions and epilepsy	37.6	53.5	29.8	4.2	32.6
COPD	71.2	48.3	33.5	15.6	12.1
Dehydration and gastro	40.8	44.8	27.5	26.0	28.2
Dental condition	35.1	51.8	31.8	11.8	27.2
Diabetes complications	44.9	54.6	30.6	15.6	9.6
Dyspepsia / otr stomach function	40.5	50.2	27.6	21.7	50.2
ENT infection	10.2	52.9	30.8	34.2	53.0
Fractured proximal femur	80.8	26.9	18.1	1.0	0.5
Hypertension	60.9	41.3	25.7	26.3	35.5
Influenza and pneumonia	67.5	51.2	24.7	17.0	7.5
Iron-deficiency anaemia	65.0	38.8	25.8	37.0	20.0
Migraine / acute headache	42.2	35.6	26.6	23.3	37.5
Neuroses	47.6	44.0	31.2	7.4	30.6
Pelvic inflammatory disease	32.6	0.0	30.7	24.5	15.1
Perforated / bleeding ulcer	56.5	54.1	26.7	19.3	24.3
Peripheral vascular disease	69.1	53.3	26.0	31.7	22.5
Pyelonephritis	63.2	34.7	24.4	19.9	15.5
Ruptured appendix	36.2	58.1	18.9	28.0	0.3
Schizophrenia	42.5	63.0	45.3	2.6	4.8
Senility / dementia	83.6	38.5	22.0	11.4	14.7
Stroke	74.7	49.3	20.0	6.7	3.7

### Association between unplanned ACSC admission rates and practice characteristics

**Fig. 1:**
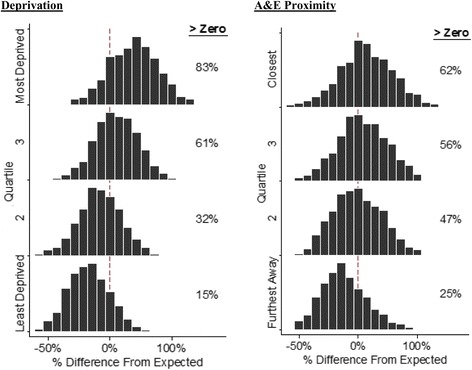
Distribution of hospital admission rates, adjusted for age and sex. *Dotted red line* represents where the number of observed admissions matches the number that would be expected given the size and age-sex composition of the practice

For all ACSCs combined, practices located in deprived areas (at the 90th centile) had 16% (95% CI: 14, 18) higher admission rates than those situated in affluent localities (at the 10th centile, Table 3). The quartile of practices closest to A&E had 8% (95% CI: 7, 10) higher admission rates than those in the furthest away quartile. Practice admission rates were highly dependent on these characteristics. For example, after adjusting for age and sex, 83% of practices based in the most deprived quartile of areas had admission rates above the national average compared to only 15% in the least deprived areas (Fig. 1). Practices with poor care continuity (10th centile) had 9% (95% CI: 8, 11) more admissions than those with good continuity (90th centile, Table 3). Practices located in PCTs with more hospital beds had higher (12%, 95% CI: 8, 17) admission rates, as did those with a larger proportion of day case admissions (17%, 95% CI: 12, 22). The association between admission rates and primary care access, clinical quality, and practice size was much smaller.

**Table 3:** Difference in unplanned admissions between a high (90th centile) and low (10th centile) practice for selected conditions^a^

	All ACSCs Combined	Alcohol- Related Diseases	COPD	Dehydration and Gastro	ENT Infections	Hypertension	Hip Fracture	Stroke
Practice characteristics								
Deprivation	16 (14,18)	44 (35,54)	36 (30,42)	8 (5,11)	7 (3,12)	7 (−5,20)	7 (3,11)	12 (8,16)
A&E Distance								
Furthest Away	Ref	Ref	Ref	Ref	Ref	Ref	Ref	Ref
2nd Quartile	5 (4, 6)	9 (3,15)	6 (2,10)	8 (5,10)	4 (0, 7)	3 (−6,12)	−1 (−3, 2)	3 (0, 5)
3rd Quartile	7 (5, 8)	15 (8,23)	10 (6,14)	9 (6,12)	4 (0, 8)	8 (−2,19)	−1 (−4, 1)	1 (−2, 4)
Closest	8 (7,10)	21 (13,29)	10 (5,14)	14 (11,17)	11 (7,16)	12 (2,24)	−3 (−6,−0)	2 (−1, 5)
Continuity	−9 (−11,−8)	−7 (−11,−2)	−13 (−16,−10)	−7 (−9,−5)	−10 (−13,−7)	−20 (−26,−13)	−0 (−3, 3)	−2 (−5, 0)
Access	−1 (−2, 0)	1 (−3, 6)	−1 (−4, 2)	−2 (−4, 0)	−0 (−3, 2)	−2 (−9, 5)	1 (−1, 4)	2 (−1, 4)
Quality	−2 (−3,−1)	−4 (−7,−1)	−1 (−3, 1)	−1 (−3, 0)	−3 (−5,−1)	−4 (−10, 2)	1 (−1, 3)	−1 (−3, 1)
Size	−2 (−3,−1)	2 (−3, 6)	0 (−3, 3)	−1 (−3, 1)	1 (−2, 4)	−1 (−8, 6)	2 (0, 4)	0 (−2, 2)
PCT characteristics								
Bed Availability	12 (8,17)	39 (21,60)	16 (9,23)	24 (16,32)	25 (12,41)	24 (9,40)	−1 (−4, 2)	5 (1, 9)
% Day Cases	17 (12,21)	12 (−3,29)	9 (4,14)	28 (20,37)	183 (149,222)	1 (−11,14)	0 (−3, 3)	2 (−1, 6)

^a^After adjustment age, sex and chronic disease prevalence (atrial fibrillation, asthma, cancer, CKD, COPD, dementia, epilepsy, heart failure, hypertension, learning disability, mental health problems, obesity and stroke) and each of the factors listed in the table

**Fig. 2:**
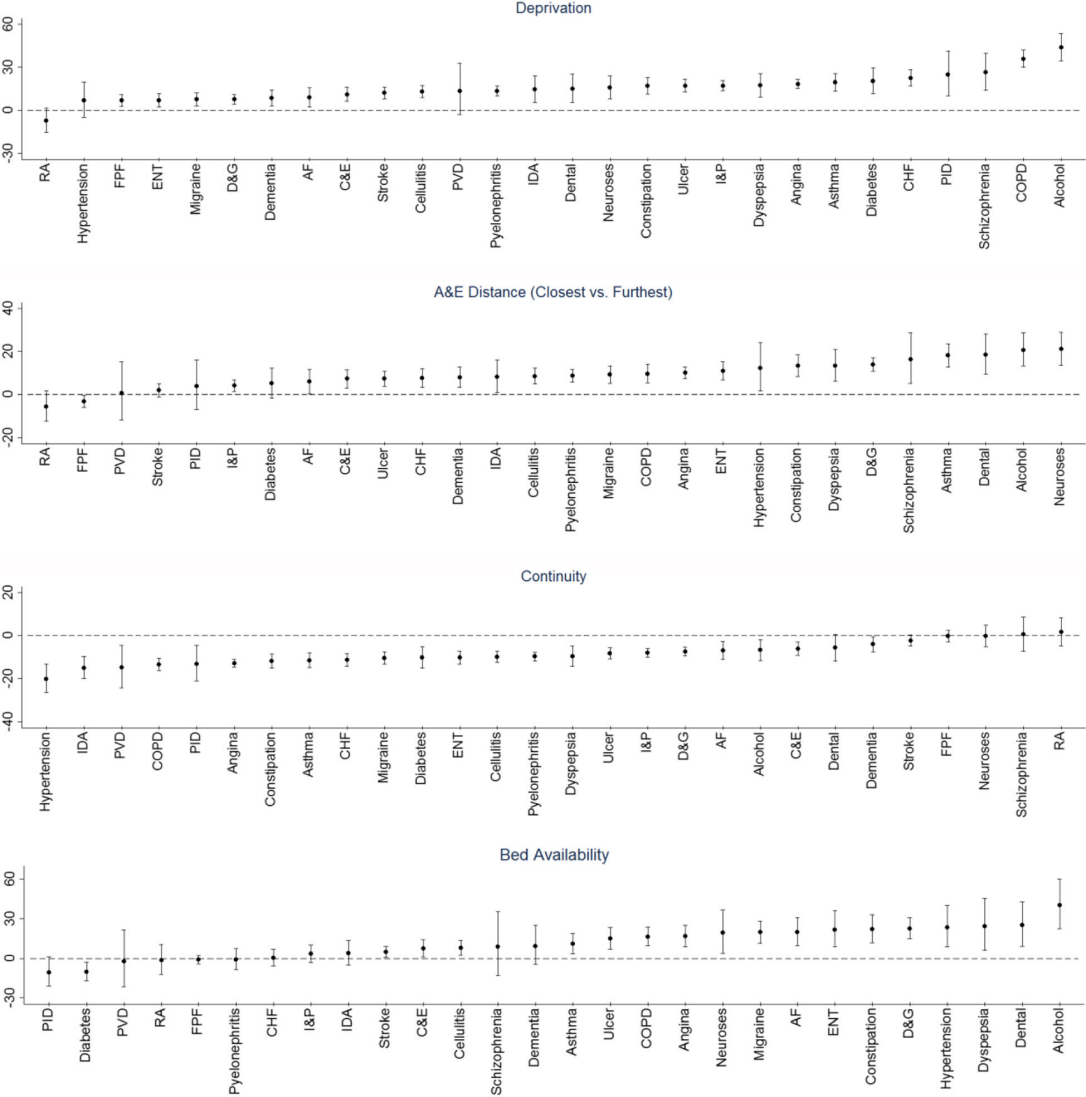
Association of unplanned admission rates with selected practice and PCT characteristics. *Circles* represent the point estimate of the relative risk between the covariate and unplanned admission rates. Vertical lines represent 95% confidence intervals

The strength of associations varied substantially across ACSCs (Table 3, Fig. 2, Additional file 4). For example, admission rates for alcohol-related diseases and COPD were more than a third higher among patients registered at practices in deprived areas while differences of less than 8% were found for several ACSCs including hypertension and migraine / acute headache. Improved continuity of primary care was most strongly associated with lower unplanned admission rates for chronic conditions such as hypertension, COPD, peripheral vascular disease and iron-deficiency anaemia. Proximity to A&E was most strongly associated with high admission rates for mental-health conditions, alcohol-related diseases, and several conditions where patients were regularly admitted for less than a day (e.g. dental conditions, asthma, constipation).

Areas with increased bed availability had particularly high admission rates amongst conditions where very short hospital stays are commonplace (e.g. alcohol-related diseases, ENT infection, dehydration and gastroenteritis). PCTs with a larger proportion of day cases had increased admission rates for most ACSCs; this was particularly pronounced for ENT infections where areas with a high proportion had 183% (95% CI: 149, 222) more admissions than those with a low proportion. Unplanned admission rates for some ACSCs appear to be much less sensitive to population, practice and hospital characteristics. For example, hip fracture and stroke admission rates had little association with continuity of GP care, A&E proximity or bed availability.

## Discussion

### Statement of principal findings

ACSCs accounted for 1.77 million admissions and 10.9 million bed days during 2011/12. Admission rates were higher in practices located in deprived areas, within close proximity to an A&E department, with poor continuity of GP care, and situated in areas with a high inpatient bed supply or proportion of day case admissions. There was little evidence that access to primary care, clinical quality, or practice size were important contributors to variation in unplanned admissions. The strength of associations differed markedly across conditions.

### Strengths and weaknesses

This study is based on a large nationally representative dataset containing almost all unplanned admissions in England. Applying standardised and robust methodology to a broad range of ACSCs provides new evidence on the factors associated with admission rates, and allows identification of the characteristics that should be prioritised when redesigning services and developing admission avoidance interventions.

Our study is observational; although we have undertaken extensive case-mix adjustment it is still possible that our results are confounded by unmeasured factors. For example, it is possible that our finding of lower admission rates among practices with higher care continuity could be due, at least in part, to these practices better organising their care in other ways (e.g. support for self-care, specialist disease clinics). However, the negligible associations for conditions where admission is not usually avoidable (e.g. hip fracture) suggests that residual confounding is not solely driving our results. Some of the characteristics considered within our study may be poorly measured. Specifically, QOF clinical scores may not accurately reflect primary care quality [13], the IMD of a practice postcode may not accurately reflect the socioeconomic status of their registered patients [14], straight line distances to A&E departments may be a poor measure of travel times [15], and our survey-based estimates of primary care access and continuity may be imprecise. This measurement error is likely to have led us to underestimate the association between unplanned admission rates and each of the variables included within our analysis due to regression dilution bias [16].

Admission rates for mental health conditions may be inaccurate [17]. Although mental health trusts report data to HES, a range of community-based alternatives (e.g. crisis houses) may substitute for hospital treatment and are not included in the dataset. Our findings of a very strong association between ENT infection admission rates and the proportion of day case admissions may be explained by variable recording of clinical decision unit activity across hospitals, or the availability of alternatives to short-stay admission (e.g. ENT ‘hot’ clinics) in some trusts [1]. Our study does not include information on patient outcomes or resource use outside hospital which prohibits identification of the ‘optimal’ admission rate.

### Comparison with other studies

Several studies have identified the crucial role that deprivation and A&E proximity play in driving unplanned ACSC admission rates [9]. There is a growing body of evidence that improved continuity of primary care is associated with lower unplanned ACSC admission rates [9]. For example, one English study found admissions reduced by 0.5% per percentage point increase in the number of patients able to book with a specific GP [18]. Strong associations between inpatient bed availability and unplanned ACSC admission rates have been detailed elsewhere [10, 19], including in a UK study which found that patients resident in areas with the highest quintile of hospital beds had 13–15% higher respiratory disease admission rates than those within the lowest quintile [19].

A recent systematic review found little evidence of reduced unplanned ACSC admission rates among practices with better appointment availability within the UK or Europe [9]. Several studies investigating the association between QOF scores and unplanned admission rates have found little or no association except for a few specific conditions (e.g. COPD, CHD) [20]. There is negligible evidence that unplanned admission rates differ by general practice size [9].

### Implications for clinicians and policymakers

Increasing unplanned admission rates are an important problem facing healthcare systems worldwide. Addressing the variance in continuity of GP care, finding and publicising convenient alternatives to admission for patients registered at practices closest to A&E, and reducing the number of short-stay admissions are potential components of successful admission avoidance interventions. Our results suggest that the success of each of these strategies is likely to vary by condition and that a multifaceted programme of interventions, tailored to local problems, is required to contain secondary care demand and reduce costs. Some of the characteristics investigated in this study are not readily modified (e.g. deprivation) or are largely beyond the control of GPs (e.g. local hospital bed availability, A&E proximity). Failure to account for these during routine audit or benchmarking could undermine efforts to detect and manage poorer quality primary care. Given the results of our study, it is likely that distinct adjustment models will be required for each condition to guard against residual confounding.

Continuity of care is an important facet of primary care, particularly for chronic conditions such as hypertension, COPD and CHF which often rely on frequent primary care management and co-occur with other morbidities. If the assocations observed in our study are causal, nearly 100,000 ACSC admissions could be avoided by practices annually through providing ‘good continuity’ care (defined being able to see or speak to their preferred GP ‘Always’,’ Almost always’ or ‘A lot of the time’ to the question) to 90% of their registered patients. Assuming an average ACSC admission cost of £1,739 [21], this would equate to hospital savings of around £170 million. Improved continuity would most obviously be attained through a growth in GP numbers, although this is likely to be challenging due to ongoing GP recruitment issues in many countries [22]. Other practice-level changes such as patient education, empowering patients to trade-off GP choice and speed of access, and better practitioner communication might be more easily achieved [23].

Our findings do not support recent UK government initiatives that focus on improvements in primary care access [24], particularly if these are implemented to the detriment of the continuity of care. A UK-based review investigating the link between primary care access and unplanned admission rates concluded that ‘Existing evidence…is inadequate to inform national policy’ [25]. Our results concur with this finding and suggest that a fuller investigation of the benefits and costs of increased access is required before implementation. The small and inconsistent association between clinical quality scores and admission rates suggests that this crude measure of primary care quality may not adequately encompass the most important aspects of GP care for averting unplanned ACSC admissions. The extent to which the care processes that the QOF incentivises lead to improvements in patient outcomes has generated substantial debate [26] and continues to evolve. The latest evidence suggests the QOF has been responsible for an 8% reduction in unplanned admission rates for incentivised ACSCs [27].

ACSC admission rates may be higher in some areas due to the increased frequency of very short admissions. Nationally, the number of short-stay admission has surged by 124% in the last 15 years [1] however our results suggest substantial geographic variation in their use. Some short hospital stays are clinically appropriate (e.g. severe exacerbations of asthma), however others may result from inappropriate referrals, limited community-based care options, patient confusion around out-of-hours care arrangements, or government targets (e.g. 4-hour A&E waits) [1]. Improved referral guidelines, enhanced community-based treatment options (e.g. telemedicine) or increased accessibility and awareness of alternatives to A&E attendance (e.g. walk-in clinics) might reduce these admissions, although there remains little research exploring their affect on overall healthcare outcomes and costs [28]. The strong influence exerted by inpatient bed availability and A&E proximity on admission rates suggests that some ACSC care may be ‘supply sensitive’ [29].

### Unanswered questions and future research

Randomised controlled trials examining the effect of new interventions to improve the continuity of primary care are required. Evaluation of community-based interventions (e.g. improved self-management) aimed at preventing unnecessary hospital admissions are needed, particularly in deprived areas and those within close proximity to A&E.

## Conclusions

Increasing unplanned hospital admissions is a key problem facing healthcare systems worldwide. The drivers of high unplanned ACSC admission rates are complex and include population (e.g. higher deprivation), practice (e.g. poor continuity of care) and hospital (e.g. increased inpatient bed availability) factors. The importance of these varies substantially across conditions suggesting multifaceted interventions are required to improve care and reduce costs. Several of the most important drivers of admissions are beyond the control of GPs. Strategies to increase the continuity of primary care and avoid unnecessary short-stay admissions could reduce admissions; however a fuller understanding of how these interventions affect aggregate healthcare costs and patient outcomes is required.

## Additional files

Additional file 1: Included ACSCs and ICD-10 codes used to define them. List of ICD-10 codes used to identify admissions for each of the conditions used within the analysis. (DOCX 39 kb)

Additional file 2: Independent variable description, potential weaknesses and source. Description of each independent variable used in the analysis. (DOCX 46 kb)

Additional file 3: Calculation of age-sex specific GP population. Description of how the age-sex specific GP practice populations were determined. (DOCX 37 kb)

Additional file 4: Association of unplanned admission rates with selected practice and PCT characteristics. Graphical representation of association between primary care access, primary care quality, practice size and percentage of day case admissions with unplanned ACSC admission rates. (DOCX 203 kb)

## Abbreviations

A&E: Accident and emergency
ACSC: Ambulatory care sensitive condition
CHD: Coronary heart disease
COPD: Chronic obstructive pulmonary disease
ENT: Ear, nose throat
HES: Hospital episode statistics
IMD: Index of multiple deprivation
NHS: National health service
PCT: Primary care trust
QOF: Quality and outcomes framework
