# Supplementary material for: How do population, general practice and hospital factors influence ambulatory care sensitive admissions: a cross sectional study
Source: BMC Fam Pract. 2017 May 25;18:67. doi: 10.1186/s12875-017-0638-9 (PMC5445441; doi:10.1186/s12875-017-0638-9)
Supplement: Supplementary file 1 — Included ACSCs and ICD-10 codes used to define them. List of ICD-10 codes used to identify admissions for each of the conditions used within the analysis. (DOCX 39 kb) [file 12875_2017_638_MOESM1_ESM.docx]

Additional file 1: Included ACSCs and ICD-10 codes used to define them

| Condition | ICD-10 Codes |
| --- | --- |
| Alcohol-related diseases | F10 |
| Angina | I20,I240,I248,I249,I25,R072,R073,R074,Z034,Z035 |
| Asthma | J45,J46 |
| Atrial fibrillation / flutter | I471,I479,I495,I498,I499,R000,R002,R008 |
| Cellulitis | I891,L010,L011,L020,L021,L022,L023,L024,L028,L029,L03,L04,  L080,L088,L089,L88,L980 |
| Congest heart failure | I110,I130,I255,I50,J81 |
| Constipation | K590 |
| Convulsions and epilepsy | G253,G40,G41,O15,R56,R568 |
| COPD | J20,J40,J41,J42,J43,J44,J47 |
| Dehydration and gastro | A020,A04,A059,A072,A080,A081,A083,A084,A085,A09,E86,K520,K521,K522,K528,K529 |
| Dental condition | A690,K02,K03,K04,K05,K06,K08,K098,K099,K12,K13 |
| Diabetes complications | E100,E101,E102,E103,E104,E105,E106,E107,E108,E110,E111,  E112,E113,E114,E115,E116,E117,E118,E120,E121,E122,E123,  E124,E125,E126,E127,E128,E130,E131,E132,E133,E134,E135,  E136,E137,E138,E139,E140,E141,E142,E143,E144,E145,E146,  E147,E148,E149 |
| Dyspepsia / otr stomach function | K21,K30 |
| Ear, nose and throat inf | H66,H67,J02,J03,J040,J06,J312 |
| Fractured proximal femur | S720,S721,S722 |
| Hypertension | I10,I119 |
| Influenza and pneumonia | A481,A70,J10,J11,J120,J121,J122,J128,J129,J13,J14,J153,J154,  J157,J159,J160,J168,J18,J181,J189 |
| Iron-deficiency anaemia | D460,D461,D463,D464,D501,D508,D509,D510,D511,D512,D513,  D518,D520,D521,D528,D529,D531,D571,D580,D581,D590,D591,  D592,D599,D601,D608,D609,D610,D611,D640,D641,D642,D643,  D644,D648 |
| Migraine / acute headache | G43,G440,G441,G443,G444,G448,R51 |
| Neuroses | F32,F40,F41,F42,F43,F44,F45,F46,F47,F48 |
| Pelvic inflammatory disease | N70,N73,N74 |
| Perforated / bleeding ulcer | K20,K210,K219,K221,K226,K250,K251,K252,K254,K255,K256,  K260,K261,K262,K264,K265,K266,K270,K271,K272,K274,K275,  K276,K280,K281,K282,K284,K285,K286,K920,K921,K922 |
| Peripheral vascular disease | I73,I738,I739 |
| Pyelonephritis | N10,N11,N12,N136,N159,N300,N308,N309,N390 |
| Ruptured appendix | K350,K351 |
| Schizophrenia | F20,F21,F232,F25 |
| Senility / dementia | F00,F01,F02,F03,R54 |
| Stroke | I61,I62,I63,I64,I66,I672,I698,R470 |
