# Supplementary material for: How do population, general practice and hospital factors influence ambulatory care sensitive admissions: a cross sectional study
Source: BMC Fam Pract. 2017 May 25;18:67. doi: 10.1186/s12875-017-0638-9 (PMC5445441; doi:10.1186/s12875-017-0638-9)
Supplement: Supplementary file 2 — Independent variable description, potential weaknesses and source. Description of each independent variable used in the analysis. (DOCX 46 kb) [file 12875_2017_638_MOESM2_ESM.docx]

Additional file 2: Independent variable description, potential weaknesses and source

| Variable | Description | Potential Weaknesses | Source |
| --- | --- | --- | --- |
| **Practice Characteristics** | |  |  |
| Deprivation | 2010 Index of Multiple Deprivation (IMD) score of the practice postcode | Deprivation of practice postcode may not adequately represent that of the practice population. | UK Data Service Census Support[[29](#_ENREF_29)] |
| A&E Distance | Straight line distance from centroid of practice postcode to closest A&E department | Straight line distances may not accurately respresent travel time, particulary in cities where travelling short distances can take a long time. | UK Data Service Census Support[[29](#_ENREF_29)] |
| Continuity | Percentage of patients who responding ‘Always’,’ Almost always’ or ‘A lot of the time’ to the question ‘How often do you see or speak to the GP you prefer?' [among those who have a preferred GP] | Survey is based on only a sample of GP patients. Question only assess relational continuity and not management or informational continuity. | GP Patient Survey[[30](#_ENREF_30)] |
| Access | Percentage of patients responding ‘On the same day’, ‘On the next working day’, or ‘A few days later’ to the question ‘How long after initially contacting the surgery did you actually see or speak to them? | Survey is based on only a sample of GP patients. | GP Patient Survey[[30](#_ENREF_30)] |
| Quality | Percentage of total clinical QOF points | QOF may not be an accurate measure of quality as it generally focuses on processes rather than outcomes, and measures care in only a subset of disease areas. | Quality and Outcomes Framework[[12](#_ENREF_12)] |
| Size | Total number of patients registered | None | Quality and Outcomes Framework[[12](#_ENREF_12)] |
| **PCT characteristics** | |  |  |
| Bed Availability | Number of overnight beds per 100,000 PCT population. Trust beds were assigned to PCTs using the Norris-Bailey proportionate flow method. Mental health beds were used for schizophrenia and neuroses, and acute beds for other conditions | Bed numbers may be incorrectly reported. Norris-Bailey method may not accurately apportion bed-days to PCTs, and may induce a positive relationship with admission rates. | NHS England Bed Availability and Occupancy Data[[31](#_ENREF_31)] |
| % Day Cases | Proportion of day case admissions | Short admissions may be recorded inconsistently across trusts, particularly in clinical decision / observation units | Hospital Episode Statistics[[32](#_ENREF_32)] |

We scaled our independent variables using the difference between a high (90^th^ centile) and low (10^th^ centile) practice to allow for easier comparison across covariates. Therefore our estimates depend on both the strength of the association and the amount of variability between practices or PCTs. There was substantial variability in almost all the practice and PCT characteristics investigated in our study (Table A1). There were very large discrepancies in the provisions of both acute (10^th^ centile: 152.1, 90^th^ centile: 264.7) and mental health (23.4, 79.0) beds per 100,000 population, and the percentage of day cases (19.8%, 31.7%). There was wider variability in primary care continuity (47.5%, 88.9%) than access (75.0%, 96.7%). There was little variation in the levels of primary care quality (as measured by QOF attainment); 90% of practices had scores between 92.8% and 100%.

Table A1: Description of practice and PCT level characteristics

| Variable | Percentile | | | | | | |
| --- | --- | --- | --- | --- | --- | --- | --- |
|  | **Min** | **10th** | **25^th^** | **50^th^** | **75^th^** | **90^th^** | **Max** |
| **Practice level characteristics** |  |  |  |  |  |  |  |
| Deprivation (IMD Score) | 0.7 | 7.1 | 12.4 | 22.2 | 38.0 | 53.7 | 85.5 |
| A&E Distance (KM) | 0.0 | 1.6 | 2.7 | 4.9 | 10.2 | 19.0 | 95.7 |
| Continuity (%) | 10.0 | 47.5 | 58.9 | 70.9 | 81.0 | 88.9 | 100.0 |
| Access (%) | 45.5 | 75.0 | 82.1 | 88.9 | 93.7 | 96.7 | 100.0 |
| Quality (%) | 16.2 | 92.8 | 96.6 | 98.7 | 99.5 | 100.0 | 100.0 |
| Practice Size (registered patients) | 1,002 | 2,346 | 3,560 | 6,042 | 9,361 | 12,434 | 44,030 |
| **PCT level characteristics** |  |  |  |  |  |  |  |
| Beds per 100,000 (Non-Mental Health) | 117.1 | 152.1 | 176.1 | 198.0 | 226.4 | 264.7 | 390.9 |
| Beds per 100,000 (Mental Health) | 0.7 | 23.4 | 32.7 | 45.0 | 59.6 | 79.0 | 191.3 |
| Day cases (%)**^a^** | 12.5 | 19.8 | 21.3 | 25.0 | 27.8 | 31.7 | 36.2 |

^a^ For all ACSCs combined. Note that the percentage of day cases varies by condition
