# Supplementary material for: How do population, general practice and hospital factors influence ambulatory care sensitive admissions: a cross sectional study
Source: BMC Fam Pract. 2017 May 25;18:67. doi: 10.1186/s12875-017-0638-9 (PMC5445441; doi:10.1186/s12875-017-0638-9)
Supplement: Supplementary file 3 — Calculation of age-sex specific GP population. Description of how the age-sex specific GP practice populations were determined. (DOCX 37 kb) [file 12875_2017_638_MOESM3_ESM.docx]

Additional file **3: Calculation of age-sex specific GP population**

As age-sex specific practice populations were not available in 2011/12 we used data from 2013/14 to calculate the proportion of the practice population in each age and gender group. We multiplied these by the practice list size in 2011/12 to estimate the age-sex specific populations. Due to practice closures and mergers these data were not available for a small number of practices (n=183; 2.3%). In these cases we estimated the age-sex composition using the populations of the five geographically closest practices where these data were available.
