# Supplementary material for: How do population, general practice and hospital factors influence ambulatory care sensitive admissions: a cross sectional study
Source: BMC Fam Pract. 2017 May 25;18:67. doi: 10.1186/s12875-017-0638-9 (PMC5445441; doi:10.1186/s12875-017-0638-9)
Supplement: Supplementary file 4 — Association of unplanned admission rates with selected practice and PCT characteristics. Graphical representation of association between primary care access, primary care quality, practice size and percentage of day case admissions with unplanned ACSC admission rates. (DOCX 203 kb) [file 12875_2017_638_MOESM4_ESM.docx]

Additional file 4: Association of unplanned admission rates with selected practice and PCT characteristics^e^

| 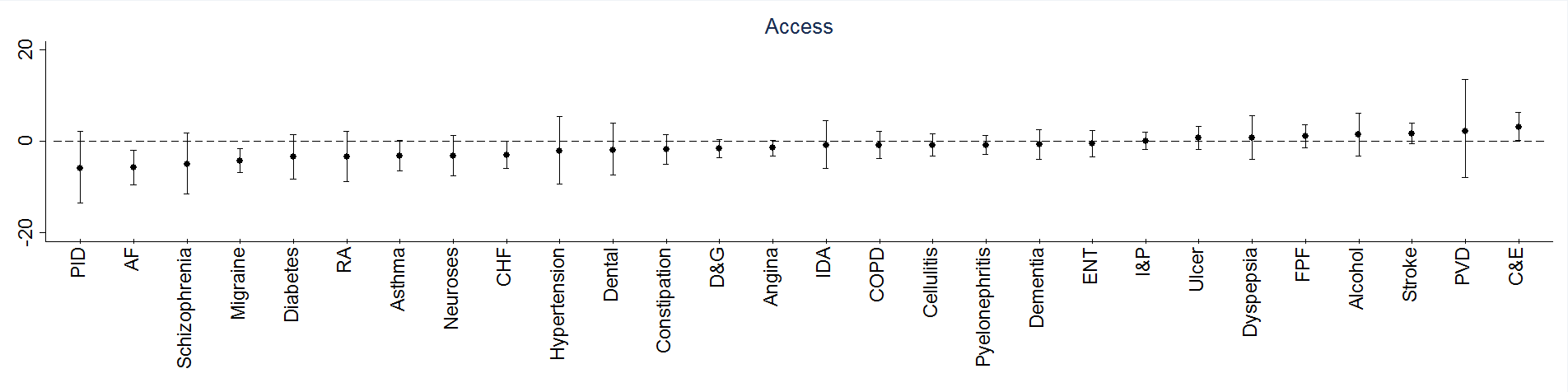 |
| --- |
| 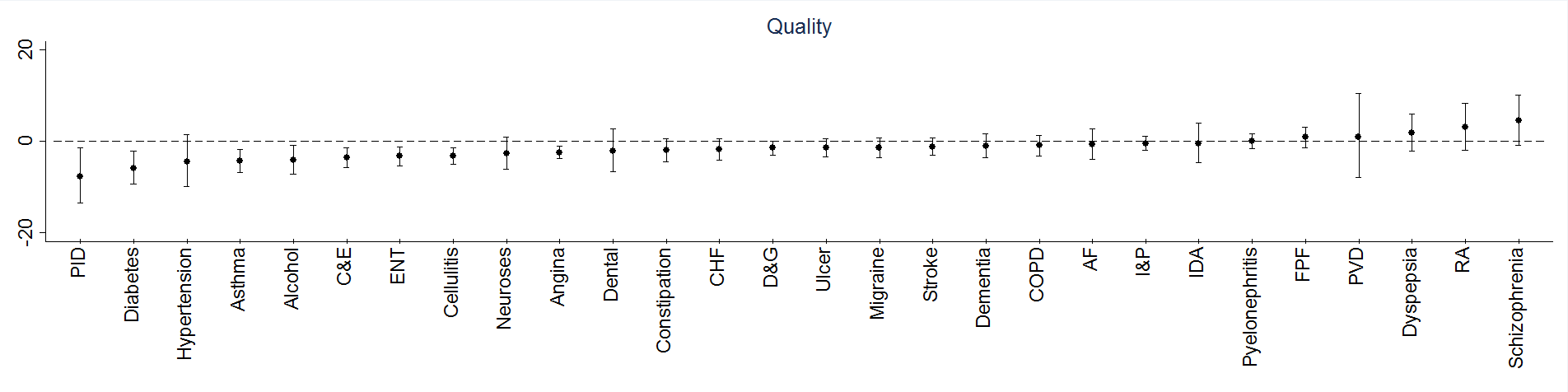 |
| 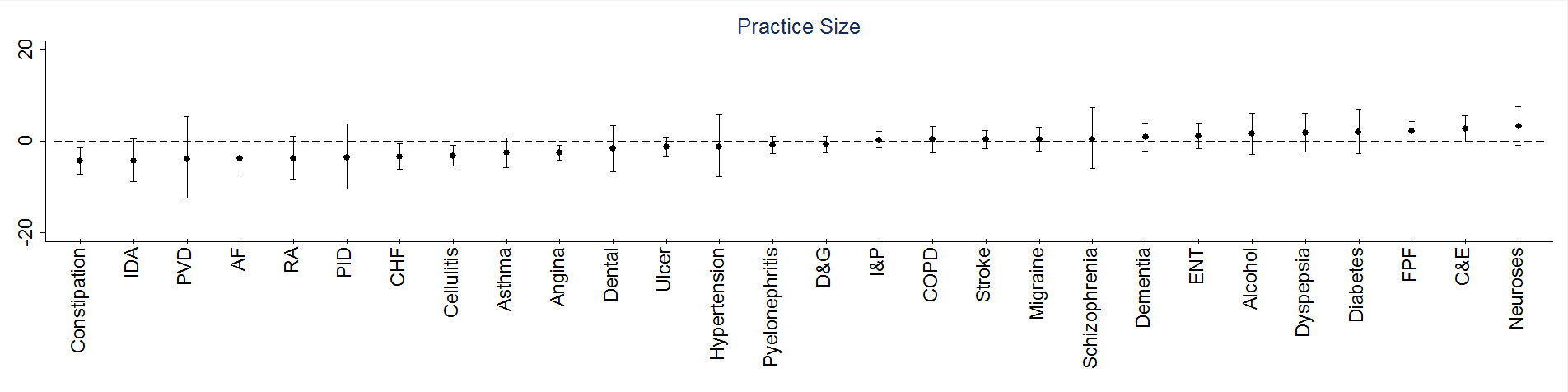 |
| 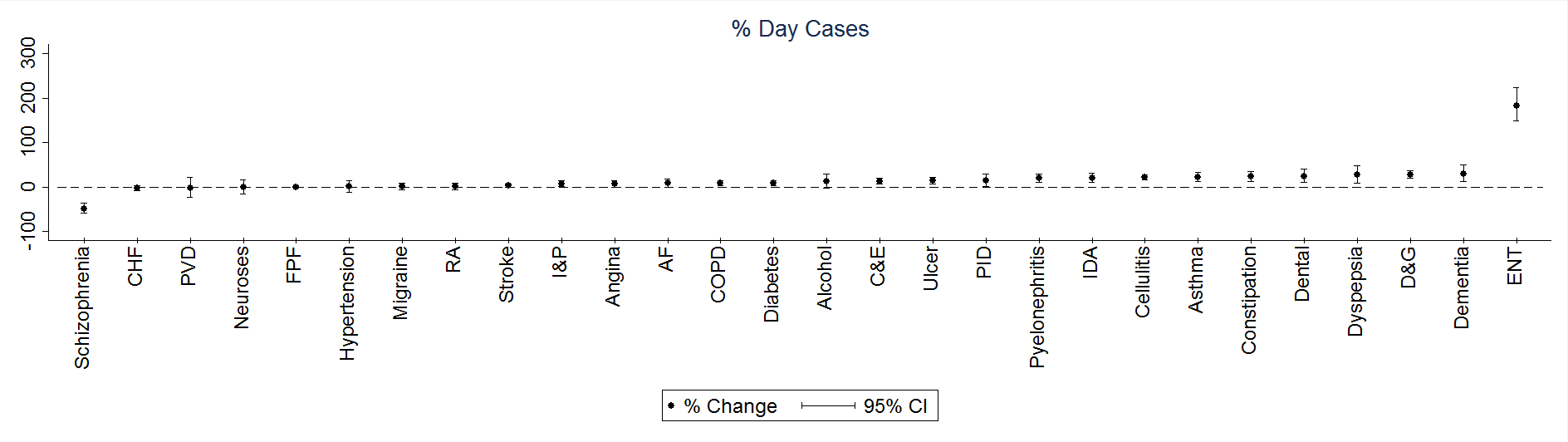 |

^e^ RA=Ruptured appendix; ENT=ENT infections; FPF=Fractured proximal femur; D&G=Dehydration and gastroenteritis; Migraine=Migraine / acute headache; Dementia=Senility / dementia; AF=Atrial fibrillation / flutter; C&E=Convulsions and epilepsy; IDA=Iron-deficiency anaemia; Dental=Dental condition; PVD=Peripheral vascular disease; Ulcer=Perforated / bleeding ulcer; I&P=Influenza and pneumonia; Dyspepsia=Dyspepsia / otr stomach function; Diabetes=Diabetes complications; CHF=Congest heart failure; PID=Pelvic inflammatory disease; Alcohol=Alcohol-related diseases
